# Outcome of medial hamstring lengthening in children with spastic paresis: A biomechanical and morphological observational study

**DOI:** 10.1371/journal.pone.0192573

**Published:** 2018-02-06

**Authors:** Helga Haberfehlner, Richard T. Jaspers, Erich Rutz, Jaap Harlaar, Johannes A. van der Sluijs, Melinda M. Witbreuk, Kim van Hutten, Jacqueline Romkes, Marie Freslier, Reinald Brunner, Jules G. Becher, Huub Maas, Annemieke I. Buizer

**Affiliations:** 1 Department of Human Movement Sciences, Faculty of Behavioural and Movement Sciences, Amsterdam Movement Sciences, Vrije Universiteit Amsterdam, Amsterdam, The Netherlands; 2 Pediatric Orthopaedic Department, University Children’s Hospital Basle, Basle, Switzerland; 3 Department of Rehabilitation Medicine, VU University Medical Center, Amsterdam Movement Sciences, Amsterdam, The Netherlands; 4 Department of Biomechanical Engineering, Delft University of Technology, Delft, The Netherlands; 5 Department of Orthopaedic Surgery, VU University Medical Center, Amsterdam Movement Sciences, Amsterdam, The Netherlands; 6 Laboratory for Movement Analysis, University Children's Hospital Basle, Basle, Switzerland; IRCCS E. Medea, ITALY

## Abstract

To improve gait in children with spastic paresis due to cerebral palsy or hereditary spastic paresis, the semitendinosus muscle is frequently lengthened amongst other medial hamstring muscles by orthopaedic surgery. Side effects on gait due to weakening of the hamstring muscles and overcorrections have been reported. How these side effects relate to semitendinosus morphology is unknown. This study assessed the effects of bilateral medial hamstring lengthening as part of single-event multilevel surgery (SEMLS) on (1) knee joint mechanics (2) semitendinosus muscle morphology and (3) gait kinematics. All variables were assessed for the right side only. Six children with spastic paresis selected for surgery to counteract limited knee range of motion were measured before and about a year after surgery. After surgery, in most subjects popliteal angle decreased and knee moment-angle curves were shifted towards a more extended knee joint, semitendinosus muscle belly length was approximately 30% decreased, while at all assessed knee angles tendon length was increased by about 80%. In the majority of children muscle volume of the semitendinosus muscle decreased substantially suggesting a reduction of physiological cross-sectional area. Gait kinematics showed more knee extension during stance (mean change ± standard deviation: 34±13°), but also increased pelvic anterior tilt (mean change ± standard deviation: 23±5°). In most subjects, surgical lengthening of semitendinosus tendon contributed to more extended knee joint angle during static measurements as well as during gait, whereas extensibility of semitendinosus muscle belly was decreased. Post-surgical treatment to maintain muscle belly length and physiological cross-sectional area may improve treatment outcome of medial hamstring lengthening.

## Introduction

Medial hamstring lengthening in children with spastic paresis (SP) is commonly performed to increase the range of motion (ROM) of the knee, and thereby the ability of the child to extend the knee during walking [1]. Medial hamstring lengthening includes lengthening of the distal semitendinosus (ST) and gracilis tendons, as well as aponeurotomy of the semimembranosus muscle [2–4]. Medial hamstring lengthening as part of single-event multilevel surgery (SEMLS) is thought to contribute to correction of flexed knee gait [4–7].

However, the success rate is limited in some patients due to side effects of surgery (e.g. increased anterior pelvic tilt, and lumbar lordosis as well as hyperextension of the knee during gait) [4, 8–10]. These side effects are thought to be a consequence of over-lengthening and weakening of hamstring muscles [4, 9, 10]. In addition, persistence of flexed knee gait or even recurrence after initial success have been described [4, 9, 11]. Until now, little is known about the effects of hamstring lengthening on muscle morphology (i.e. muscle belly length, tendon length and muscle volume). Detailed insight in post-surgery adaptation and function of hamstring muscles around the knee may help to increase our understanding how effects of surgery on gait, and underlying musculoskeletal structures and functions are related. Such knowledge may provide indications for improvement of surgical intervention in children with SP.

The ST, one of the hamstring muscles, is presumed to contribute to ROM limitation of the knee in children with SP and its tendon is, therefore, frequently lengthened [2–4]. In children with SP, ST has been shown to have a shorter muscle belly and lower volume than in typically developing children [12–16]. Also stiffer fascicles, sarcomeres that operate at higher length, and lower muscle fiber cross-sectional area compared to typically developing children have been reported [17]. For the medial gastrocnemius muscle it has been shown that one year after aponeurotomy, fascicles as well muscle belly length were shorter, without a loss of muscle volume [18, 19]. How morphology of ST changes in response to surgical lengthening of the distal tendon is yet unknown.

The aims of this study were to assess the effects of medial hamstring lengthening on (1) knee joint mechanics (i.e. popliteal angle, minimal knee angle towards extension and knee moment-angle characteristics) (2) ST morphology and (3) gait kinematics. We hypothesized that a longer and more compliant tendon of ST after surgery would lead to a shift in knee moment-angle curve and a decreased popliteal angle, as well as a decrease in the slope of the knee moment-angle curve. As a consequence, the knee angle in mid-stance and terminal swing of gait would be more extended.

## Methods

The study was approved by the Medical Ethics Committees of the VU University Medical Center (VUmc), Amsterdam (The Netherlands) and of the University of Basel Children’s Hospital (UKBB), Basel (Switzerland) and was registered in the Dutch and German trial register (NTR3042; DKRS00004723). All children and their parents gave written informed consent.

### Study design

In this prospective observational cohort study, we included children with SP who were scheduled for orthopaedic surgical lengthening of the muscle-tendon unit (MTU) of the medial hamstrings. Surgery was performed to increase the knee passive ROM and thereby improve gait function. Inclusion and exclusion criteria have been described recently [16] (for details, see S1 Text). All measurements were planned before first surgery and 12 months after hamstring lengthening surgery. Knee moment-angle characteristics and muscle morphology measurements were planned at six weeks, six months and 24 months after first surgery.

### Medial hamstring lengthening

All children included were scheduled to undergo medial hamstring lengthening within a SEMLS or as a single procedure. Medial hamstring lengthening was performed distally by lengthening the ST and gracilis tendons by Z-plasty and the semimembranosus muscle by aponeurotomy [20]. In one of the two centers participating in the study (UKBB), it is common practice to perform the surgical medial hamstring lengthening three months prior to final SEMLS procedure [2]

### Participants

Children were included between September 2011 until May 2015 in the VUmc and UKBB. Initially, nine children (five females and four males; six children in the VUmc and three children in the UKBB) with a mean age of 14.1±2.7 years were included in the study [16]. From these nine children, three children (two females (UKBB) and one male (VUmc) were not included in the follow-up measurements and thus in the analysis of this study. The reasons were: one child eventually was not treated by surgically lengthening of the medial hamstring but was treated by distal femoral anterior guided growth (hemi-epiphysiodesis) combined with Botulinum toxin A injections into the hamstring muscles. Another child did not tolerate ultrasound measurements due to anxiety and unrest. For the third child there were planning issues. For three of the six children included in the follow-up measurements medial hamstring lengthening of the right leg was combined with hemi-epiphysiodesis, in two others with supracondylar extension osteotomy and one child did not have additional bony procedures of the right femur. For two of the six children, serious adverse events occurred after the surgery: (1) neuropathic pain after epidural pain management (subject 1) and (2) stage IV pressure ulcer (subject 6). The exact surgical procedures, adverse events as well as the rehabilitation program are described in supporting information (S1 Table).

### Measurements

#### Patient characteristics and anthropometrics

Functional mobility level was classified by the Gross Motor Function Classification System (GMFCS) [21]. Body mass and body height were measured and body mass index (BMI) was calculated.

#### Knee joint characteristics

Knee joint characteristics were determined for the right leg. The popliteal angle was measured according to Reimers [22]. The passive knee angle with the hip in 0° was measured using the neutral zero method [23]. For knee angle measurements, neutral position was defined as 0° with increasing knee angle towards knee flexion.

Knee moment-angle characteristics were measured at rest by instrumented hand-held dynamometry. The experimental setup and procedures have been described in detail previously [16, 24]. In brief, children were positioned on their left side, with the hip of the right (measured) leg at 70° flexion. The right lower leg was positioned on a low-friction moveable plate. The knee moment was assessed at various knee angles in steps of 5°. Knee moment and knee angles were measured only when muscle activity levels measured by surface electromyography of biceps femoris, gastrocnemius medialis, rectus femoris, and vastus lateralis muscles were absent or very low [16, 24]. Data were fitted by third order polynomial functions. Knee angles at 0, 0.5, 1, 2, 3 and 4 Nm were derived from the fitted curves [16, 24]. Range of knee angles between 0 and 4 Nm was calculated and referred to as range of motion (ROM_0-4Nm_).

#### Measurements and analysis of morphological characteristic*s*

Muscle morphology of right ST was determined by freehand three-dimensional ultrasound (3D US). 3D US measurements, reconstruction and analysis method have previously been described in detail [16, 25]. US imaging was performed at three knee angles (i.e. at 65° and angles corresponding to 0 and 4 Nm knee moment).

Voxel arrays were anonymized for subjects, follow-up moment as well as for measurement condition (i.e. 0 Nm, 4 Nm, 65°) and analyzed in randomized order. To characterize ST morphology, (1) muscle-tendon unit length (ℓmtu), (2) muscle belly length (ℓm), (3) tendon length (ℓt_dist_) and (4) muscle belly volume were assessed. All length variables have been expressed as percentage of femur length (_norm_ %femur). Changes in ℓm_norm_ and ℓt_dist_norm_ from 0 to 4 Nm knee flexion moment were calculated (Δℓm_norm_, Δℓt_dist_norm_).

#### Gait kinematics

Kinematic parameters were obtained by three-dimensional instrumented gait analysis. For gait analysis in VUmc, the methods have been described in detail previously [26]. In brief, a technical clusters of three markers were rigidly attached to the body segments and anatomically calibrated by probing bony landmarks. Segment movements were tracked using an optoelectronic motion capture system (Optotrak 3020, Northern Digital, Canada). The strides were analyzed using custom-made software (Bodymech, www.bodymech.nl). Joint and segment kinematics were calculated according to International Society of Biomechanics (ISB) anatomical frames [27]. One post-operative gait analysis was analyzed by a custom-made, open-source software package to simultaneously observe gait parameters and video recordings (the MoXie Viewer^®^, http://moxie.smalll.eu/). Details have been described previously [28]. For UKBB, reflective markers (14 mm diameter) were attached bilaterally to bony landmarks on the skin. The Helen Hayes Marker set [29] was used to model the lower body. Movements of the subjects were tracked by a VICON motion capture system (twelve MXT20 cameras, 200 Hz; Vicon. Oxford, UK). VICON-software was used for the pre-processing of the data.

Joint angles were time normalized to a gait cycle, defined as the time between two consecutive foot strikes of the same leg. The parameters of interest were segment/joint angles in the sagittal plane at mid-stance (30% gait cycle) and at terminal swing (99% gait cycle): pelvic tilt, hip angle and knee angle of the right leg.

### Data treatment and statistics

Follow-up measures–scheduled six weeks after surgery–could only be performed in four of the six children and were delayed. Also other scheduled measurements were postponed or canceled (for details see S2 Table). Due to these missing data, follow-up measurements were grouped in two time windows: (1) short-term follow-up (11–20 weeks after surgery) and (2) medium-term follow-up (8–20 months after surgery). Short-term follow-up for knee moment-angle characteristics and muscle morphology measurements were only assessed in four children. As subject 1 could not walk after surgery, no gait kinematics of this subject were included.

Paired T-tests were used to test for differences between baseline and the medium-term follow-up measures of anthropometric parameters, morphological characteristics at 65° knee angle, Δℓm_norm_, Δℓt_dist_norm_, muscle volumes and gait kinematics. To test for differences in morphological characteristics (at 0 and 4 Nm) at baseline and medium-term follow-up, repeated measures two-way ANOVA (factors: time x moment) was used. Differences in knee moment-angle characteristics (0, 0.5, 1, 2, 3 and 4 Nm) at the different time points were tested using repeated measures two-way ANOVA (factors: time x moment) with knee angle as independent factor. Differences in ROM_0-4Nm_ were tested by paired T-tests. Correlations were calculated by Pearson correlation coefficient (Pearson’s r). Normal distribution was tested by Shapiro-Wilk test. If data were not normally distributed, which was the case for ℓt_dist_^*65deg*^_norm_ and muscle volume, non-parametric Related Samples Wilcoxon Signed Rank tests were used. For ANOVA, Greenhouse Geisser correction was used when the assumption of sphericity was violated. Data were presented as means±standard deviation (SD). The level of significance was set at 0.05.

## Results

### Patient characteristics and anthropometrics

Three boys and three girls were included in the follow-up measures with a mean age of 13 years and 10 months (Table 1). From baseline to the medium-term follow-up measurements, body height and body mass increased by 6.0±4.3 cm and 4.2±2.5 kg, respectively (p = 0.019, p = 0.008, Table 1), while BMI and femur length of the right leg did not change (p = 0.631, p = 0.580, Table 1). After surgery, in all children GMFCS level remained unchanged except for one child. In this child (subject 1), GMFCS increased from level III to IV (Table 1).

**Table 1:** Clinical characteristics before and 8–20 months after medial hamstring lengthening. Values are mean±standard deviation (range).

	Baseline	Medium-term follow-up (8–20 months)	p
Age (years)	13.8±2.7 (10.6–17.3)	14.8±2.8 (11.5–18.1)	
Gender (female/male)	3/3		
GMFCS	2 x II; 4 x III	2 x II, 3 x III, 1 x IV	
Body height (cm)	152.2±13.2 (136.0–176.0)	158.2±11.9 (144.0–179.0)	0.019
Body mass (kg)	46.5±11.2 (27.0–61.0)	50.8±11.5 (31.0–66.0)	0.008
Femur length (cm)	35.3±3.6 (31.6–41.0)	35.5±3.6 (32.0–41.0)	0.580
BMI	19.8±2.9 (46.6–23.2)	20.1±2.9 (15.0–23.9)	0.631

Gross Motor Function Classification System (GMFCS); BMI = Body mass index,

### Knee joint characteristics

Popliteal angle of the right leg decreased from 72±8° (range 60–80°) at baseline to 47±15° (range 30–70°) at medium-term follow-up (p = 0.017). Minimal passive knee angle towards extension of the right leg measured with the hip at 0° decreased from 29±12° (range 15–45°) at baseline to 10±11° (range 0–25°) at medium-term follow-up (p = 0.024).

**Fig 1:**
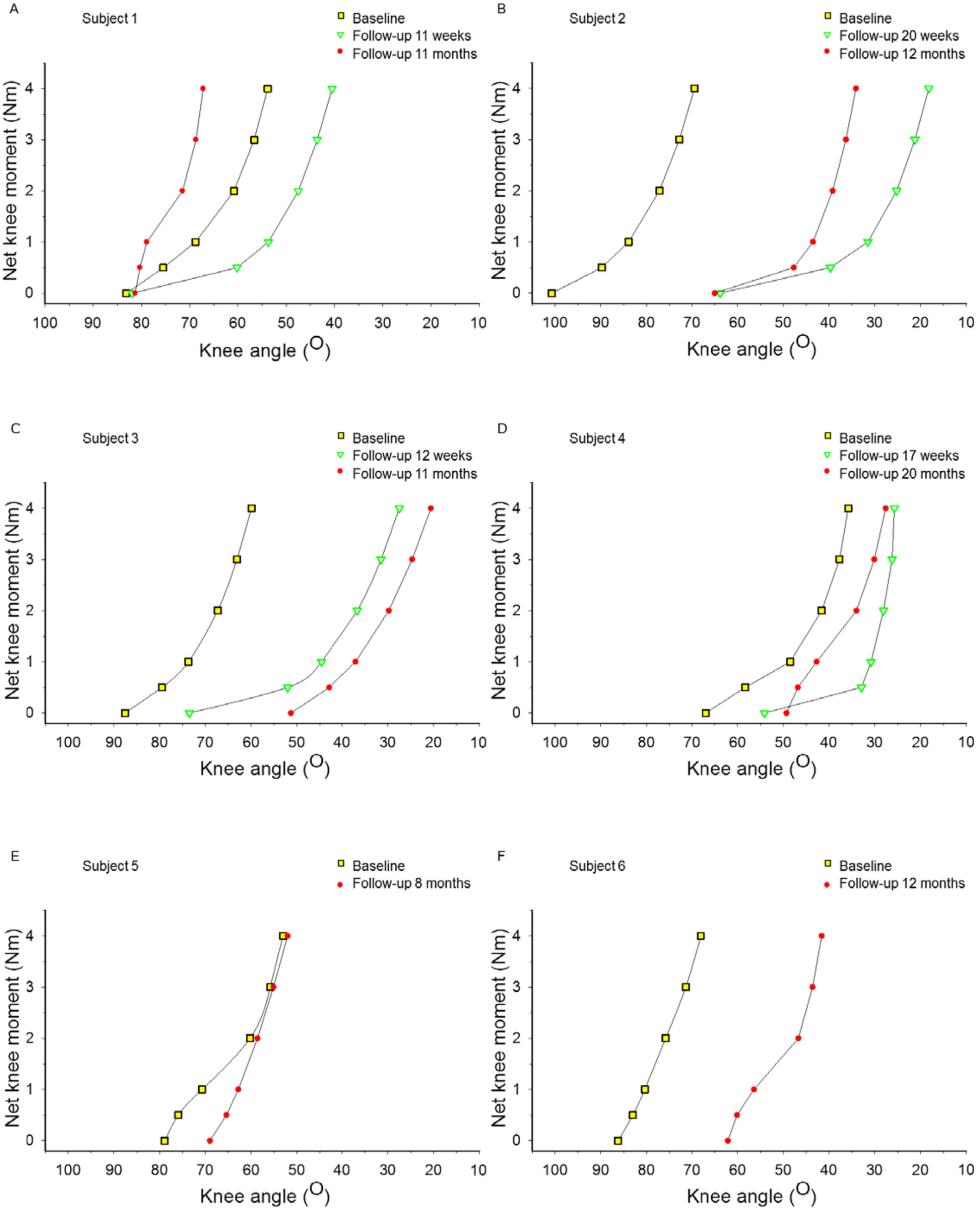
Individual net knee moments as a function of knee angle of all six subjects. A: Subject 1; B: Subject 2; C: Subject 3; D: Subject 4; E: Subject 5; F: Subject 6; Squares (yellow): Baseline; Triangle (green): short- term follow-up 11–20 weeks after surgery (for 4 subjects A-D); Dots (red): Follow-up 8–20 months after surgery.

For the four subjects (subject 1–4) for whom short-term follow-up measurements (i.e. 11–20 weeks after surgery) were obtained, ANOVA of the knee angle did not reveal any effect of time (p = 0.065). However, a significant interaction effect between time and net knee flexion moment was found (p = 0.046), indicating a change in the shape of the curve. This is most evident at low knee moments (between 0 and 1 Nm) at which the slope was less steep (Fig 1A–1D). The ROM_0-4Nm_ was 29±2° (range 27–31°) at baseline and 40±8° (range 28–46°) at short-term follow-up, but this difference was not significant (p = 0.100).

Medium-term follow-up measurements (8–20 months post-surgery) of knee moment-angle were assessed for the whole group of children. At baseline, knee angles ranged from 83±11° at 0 Nm knee flexion moment to 56±3° at 4 Nm. After surgery, knee angles ranged from 63±12° at 0 Nm net knee flexion moment to 41±17° at 4 Nm. Note that the variation in effect of surgery on the knee moment-angle curve was substantial (Fig 1). Repeated measures ANOVA revealed for the medium-term follow-up no effect of time on the knee angle (p = 0.080) and no interaction effect of time and net knee flexion moment (p = 0.768). The lack of interaction effect was supported by the observation that pre and post-surgery knee ROM_0-4Nm_ did not differ (Baseline: 28±5°; medium-term follow-up: 22±7°; p = 0.180). At medium-term follow-up, knee moment-angle curves for all subjects, except for one (subject 1) were shifted towards lower knee angles (i.e. a more extended knee, Fig 1). The largest curve shifts occured in children who had more flexed knees (i.e. higher knee angles) prior to the surgery (i.e. subject 2, Fig 1B; subject 3, Fig 1C and subject 6, Fig 1F).

For four subjects (subject 1–4), we could make comparisons between short-term and medium-term effects. No effect of time (p = 0.367), but a significant interaction effect between time and net knee flexion moment was found (p = 0.001). The knee ROM_0-4Nm_ in these four subjects changed from 40±8° at short- term follow-up to 24±9° at medium-term follow-up (p = 0.033), thus, back to baseline levels. These results indicate that at short-term folllow-up, mean slope of the knee moment-angle curve was decreased. However, at medium-term follow-up mean slope returned back towards baseline values.

### Morphological characteristics of semitendinosus muscle

**Fig 2:**
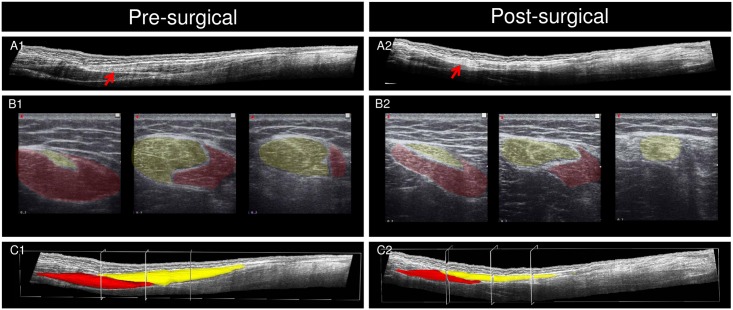
Typical example of 3D ultrasound images and segmentation of muscle volume of a child with a spastic paresis before medial hamstring lengthening. Images before surgery (left, A1-C1) and 12 months after surgery (right, A2-C2). A: longitudinal view of semitendinosus muscle (ST) (proximal on the left side); B transversal view of ST at three locations (most proximal on left side; orientation is medial-lateral). Yellow: distal compartment of ST; red: proximal compartment of ST. C: Proximal (red) and distal (yellow) compartments after segmentation. After surgery, this child showed a reduction of muscle volume by 26%, muscle belly length decreased by 32% and tendon length increased by 62%, measured at a knee angle corresponding to 4 Nm knee moment. Note the post-surgical increase in ultrasound echo intensity (cf. A2 and A1), which complicated exact identification of structures, in particular the distal and proximal ends of the tendinous inscription. The tendinous inscription is indicated by a red arrow in A1 and A2.

At baseline, we measured ℓmtu, ℓm, ℓt_dis_, muscle volume, fascicle length and volumes of the both ST compartments [16]. After surgery, however, fascicle length and proximal and distal muscle volume could only be estimated in two subjects because enhanced ultrasound echo intensity was leading to an inaccurate identification of tendinous inscription (see Fig 2 for an example of decreased image quality after lengthening of ST tendon).

**Fig 3:**
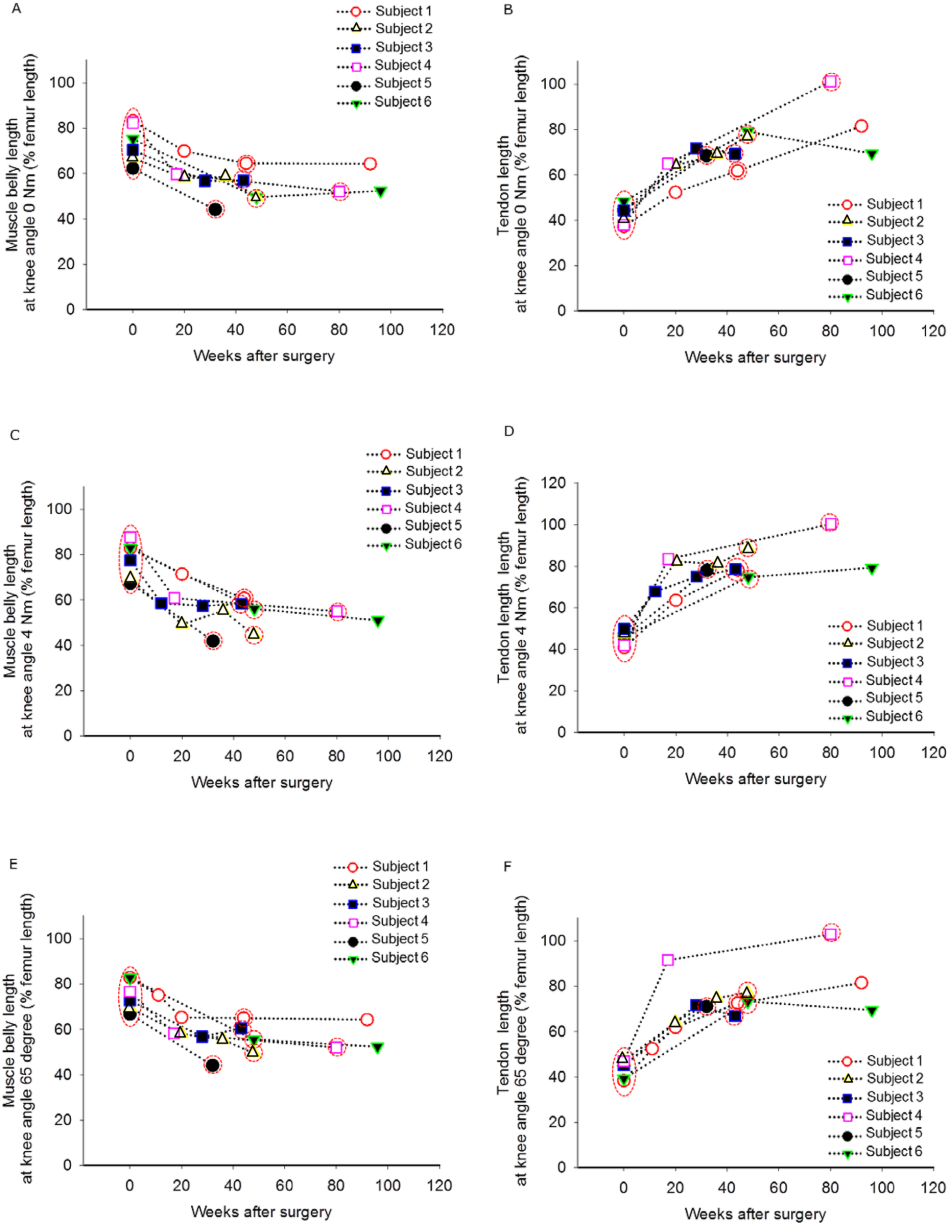
Individual effects of hamstring surgery on ST morphology. Muscle belly length and tendon length were measured at knee angles corresponding to 0 Nm (A, B), 4 Nm (C, D) and at 65° knee flexion angle (E, F). Muscle belly decreased after surgery, while tendon length increased. Time points used for statistical analysis are highlighted by red circles.

Origin and insertion distance of ST muscle at a knee angle of 65° (ℓmtu^*65deg*^_norm_) increased by 11±8% between baseline and medium-term follow-up measurement (Table 2). This increase was likely due to effects of bone surgery (i.e. hemi-epiphysiodesis and supracondylar extension osteotomy). Despite this increase in MTU length, muscle belly length at 65° knee angle (ℓm^*65deg*^_norm_) was decreased by 28±7%. In contrast, ℓt_dist_^*65deg*^_norm_ was increased by 77±26% after surgery (Table 2, Fig 3E & 3F).

**Table 2:** Morphological characteristics of semitendinosus muscle before and 8–20 months after medial hamstring lengthening (length variables as percentage of femur length (%femur) at 0 Nm (^*0Nm*^) and 4 Nm (^*4Nm*^) knee moment and 65 degree (^*65deg*^) knee angle.

	Baseline	Medium- term follow-up (8–20 months)	p
ℓmtu^*0Nm*^_norm_	115.6±7.1%femur	129.0±13.3%femur	0.020
ℓmtu^*4Nm*^_norm_	123.3±6.8%femur	135.8±11.6%femur	
Δℓmtu_norm_	7.7±3.4%femur	6.8±4.4%femur	0.719
ℓmtu^*65deg*^_norm_	118.9±4.3%femur	131.8±13.4%femur	0.025
ℓm^*0Nm*^_norm_	73.4±8.5%femur	52.8±7.1%femur	0.000
ℓm^*4Nm*^_norm_	78.0±8.1%femur	52.8±7.7%femur	
Δℓm_norm_	4.6±3.0%femur	-0.1±4.4%femur	0.008
ℓm^*65deg*^_norm_	75.2±6.8%femur	54.4±7.6%femur	0.000
ℓt_dist_^*0Nm*^_norm_	42.2±4.3%femur	76.2±13.8%femur	0.001
ℓt_dist_^*4Nm*^_norm_	45.3±3.4%femur	83.0±9.6%femur	
Δℓt_dist_norm_	3.1±3.1%femur	6.8±7.8%femur	0.216
ℓt_dist_^*65deg*^_norm_	43.7±3.9%femur	77.3±12.9%femur	0.028
Muscle volume	34.8±19.7 cm^3^	19.5±16.1 cm^3^	0.058

ℓmtu = length of muscle-tendon unit as the sum of ℓm and ℓt_dist_; ℓm = length muscle belly: ischial tuberostiy distal muscle tendinous junction; ℓt_dist_ = length of distal tendon; all length variables were expressed as % of femur length (_norm_).

After surgery, ℓmtu_norm_ measured at net knee flexion moments of 0 Nm and 4 Nm were increased (i.e. 12±9% longer at 0 Nm and 10±7% at 4 Nm, Table 2). Muscle belly lengths (ℓm_norm_) at 0 Nm and 4 Nm were decreased after surgery by 28±7% and by 33±6%, respectively (Table 2; Fig 2A & 2C). Tendon length at 0 Nm (ℓt_dist_norm_) was increased by 83±43% and by 85±30% at 4 Nm (Table 2; Fig 2B & 2D). For ℓm_norm_, a significant interaction effect was found between factors time (i.e. baseline and medium-term follow-up) and knee moment (p = 0.008). However, no such effect was found for ℓmtu_norm_ and ℓt_dist_norm_ (p = 0.719 and p = 0.216, respectively). These interaction effects were also indicated by a smaller Δℓm_norm_ at medium-term follow-up compared to that at baseline, while Δℓmtu_norm_ and Δℓt_dist_norm_ did not differ significantly (Table 2).

In four of the six subjects muscle volume of ST decreased substantially between baseline and medium-term follow-up measurements. In one subject muscle volume decreased slightly and in one subject muscle volume was slightly increased (S2 Table). Overall an average 44% decrease was found, however this decrease did not reach significance (p = 0.075, Table 2). The more pronounced decrease in muscle volume after medial hamstring lengthening compared to the decrease in muscle belly length, suggests that at medium-term follow up the physiological cross-sectional area (PCSA) of ST had reduced.

In successive follow-ups, for ℓm or ℓt_dist_ no changes towards values measured before surgery could be shown (Fig 3). Therefore, the observed recurrence of the steepness of the knee moment-angle curve between short-term and medium-term follow-up (Fig 1) does not seem to be explained by changes in ST morphology.

### Gait kinematics

**Fig 4:**
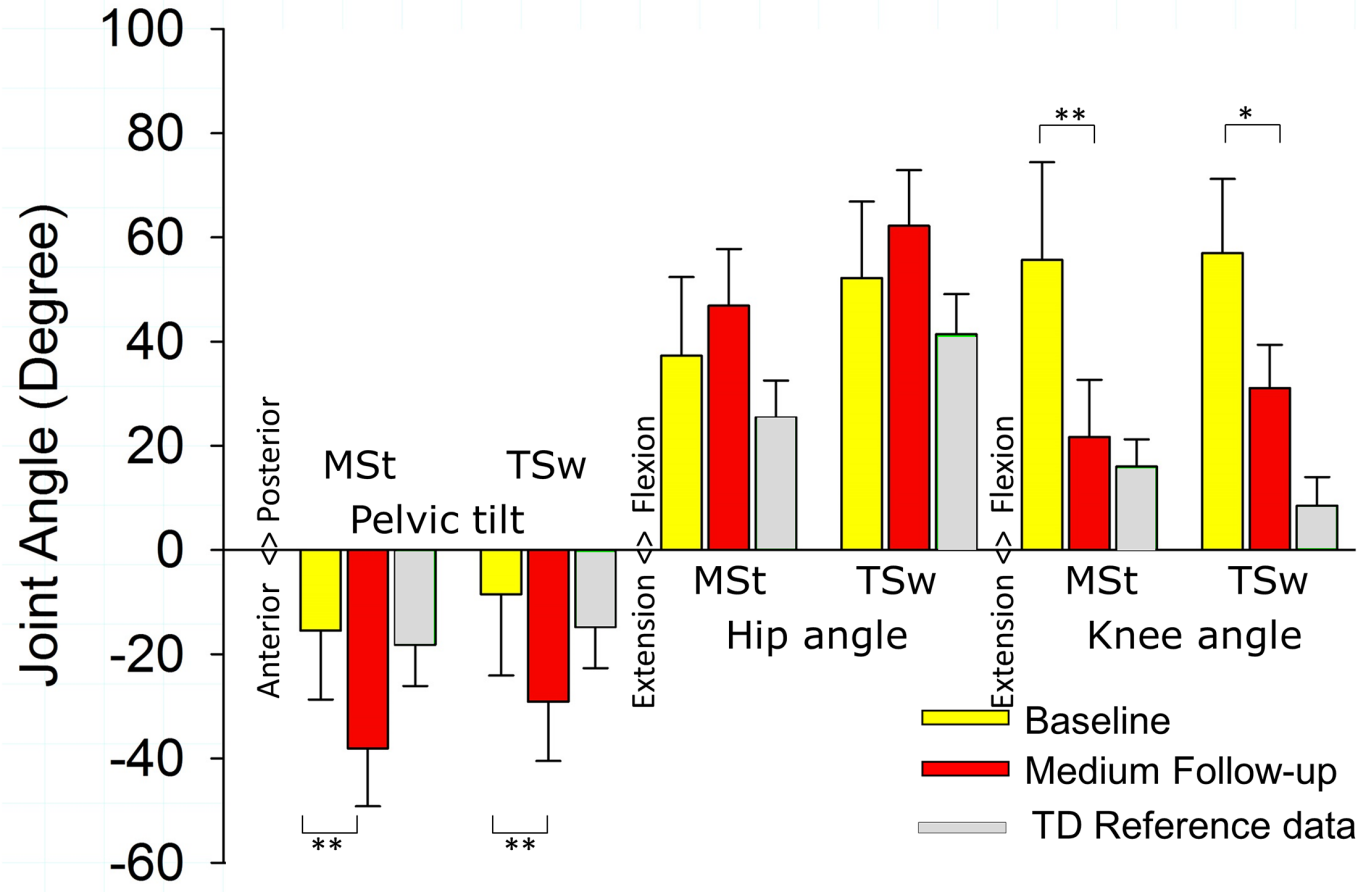
Effects of hamstring muscle surgery on gait kinematics. Presented data were measured before (Baseline, yellow) and 8–20 months (Medium follow-up, red) after medial hamstring lengthening. In grey the reference data of a group of typically developing children (TD) are presented. Values are mean±standard deviation. MSt = Mid-stance; TSw = Terminal Swing. **p<0.01; *p<0.05.

**Fig 5:**
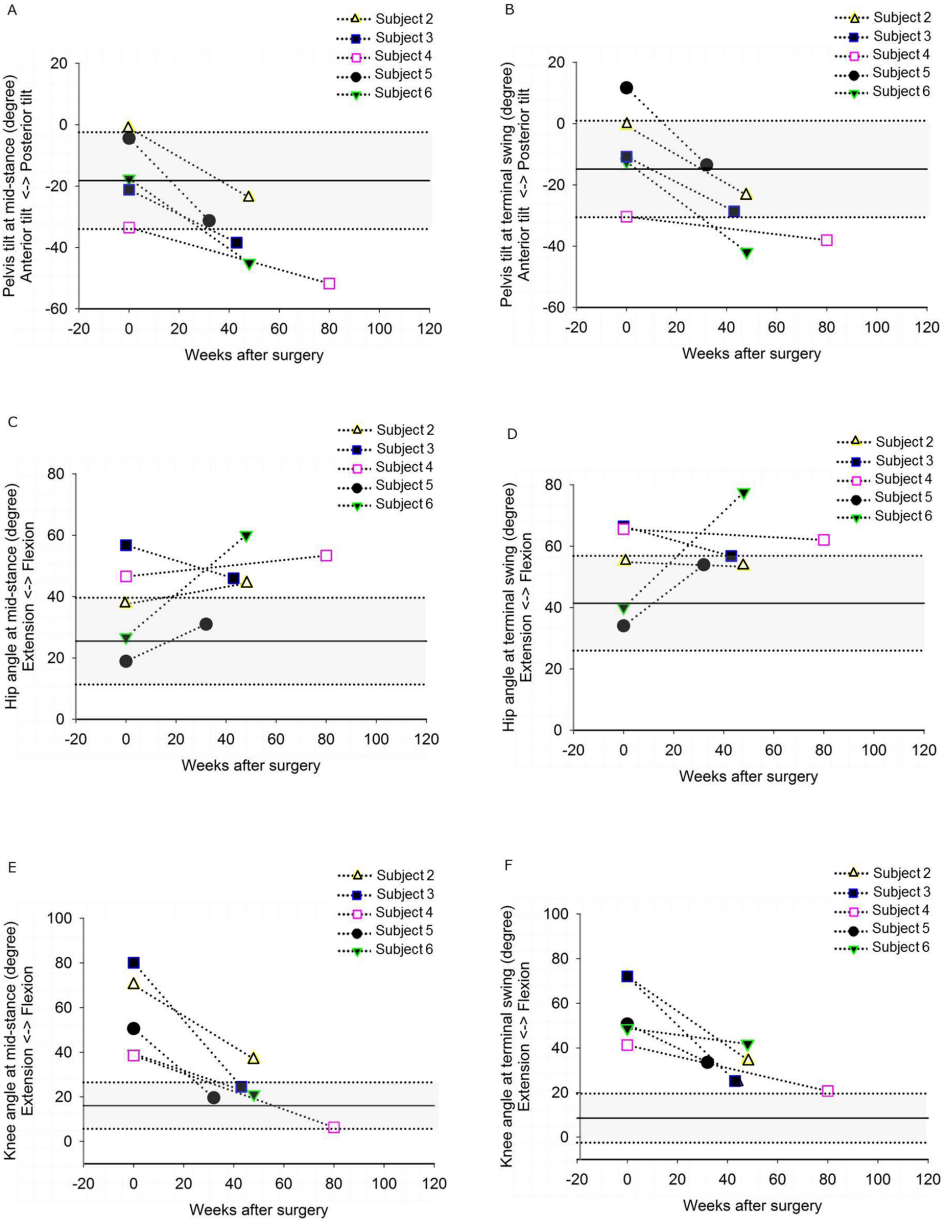
Individual gait kinematics before and after hamstring lengthening surgery. Pelvic tilt at mid-stance and terminal swing (A, B), hip angle at mid-stance and terminal swing (C, D) and knee angle at mid-stance and terminal swing (E, F) at baseline and 8–20 months after surgery. Pelvic tilt changed towards more anterior tilt and knee joint was more extended both at mid-stance and terminal swing, while effects on hip angles were variable. The grey area represents the mean and two standard deviations of a group of typically developing children. Note that most of the children used a walking device during gait analysis, which most likely contributed to the anterior pelvic tilt. In addition, during gait analysis three subjects (subjects 3, 4 and 6) walked with different support after surgery than before (see S3 Table).

**Fig 6:**
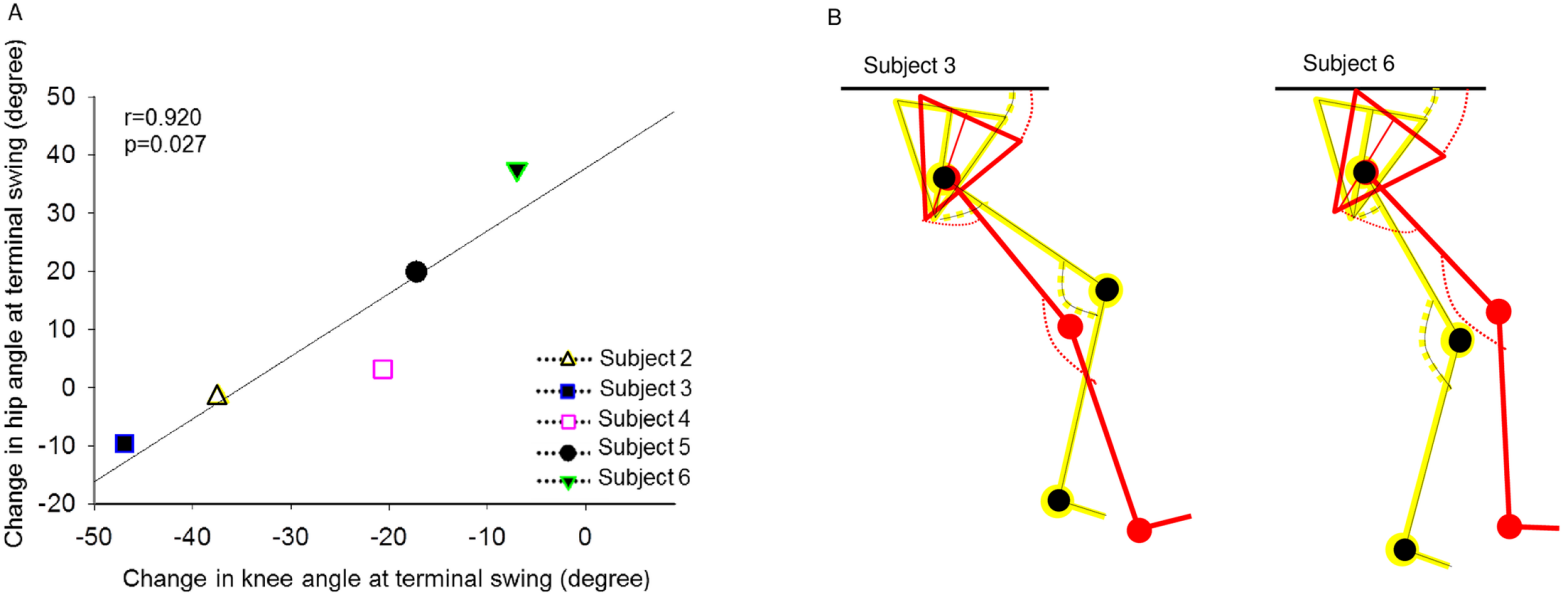
Relation between change in knee angle at terminal swing and change in hip angle at terminal swing. A: Regression analysis showed a significant relation. The more extended knee joint during terminal swing and the change in hip angle (r = 0.920; p = 0.027), indicating that the children with larger improvements of knee angles (i.e. more extended knee joint) showed larger decreases in hip flexion. B: Typical stick diagram of two subjects for hip and lower leg joint angles before and after surgery. Yellow represents the baseline (pre-surgical) of joint angles and pelvic tilt during terminal swing and red represents medium follow-up. Anterior pelvic tilt increased in both subjects after surgery. In subject 3, hip angle flexion decreased by 10° and knee joint angle towards extension increased substantially (47°), while in subject 6, hip flexion angle increased substantially (i.e. 38°) and knee joint angle towards extension increased only slightly (7°).

At baseline and medium-term follow-up, sagittal plane joint kinematics could be assessed in five out of six children (S3 Table). The knee was more extended during mid-stance and terminal swing, but pelvic anterior tilt was increased (Fig 4). While in all children, during mid-stance and terminal swing the knee was more extended and pelvic anterior tilt was increased, effects on the hip angle were variable. The hip was more extended in one child (subject 3), slightly more flexed (about 10°) in three children (subject 2, 4 and 5) and substantially more flexed in one child (subject 6) (Fig 5). A more extended knee joint during mid-stance and terminal swing correlated positively with a decrease in hip angle (i.e. more extended hip joint) (r = 0.954; p = 0.012 and r = 0.920; p = 0.027), suggesting that children with less improvement towards knee extension showed a higher increase in hip flexion (Fig 6).

## Discussion

This study shows that after medial hamstring lengthening popliteal angle and minimal passive knee joint angle towards extension were both improved. In most subjects, knee moment-angle curves initially showed a shift towards more extended knee angles, while knee ROM between 0 and 4 Nm knee flexion moment increased, but recurred to pre-surgery values at medium-term follow-up. Muscle belly length decreased while tendon length increased and the change in muscle belly length from 0 to 4 Nm decreased. In gait, knee joint angles measured at medium-term follow-up were more extended in mid-stance and terminal swing, but pelvic anterior tilt increased also. There was no consistent change in hip angle. Individual data indicate that less improvement of the knee joint angles towards extension angles during gait correlated with more increase of hip flexion.

### Changes in knee joint characteristics and ST morphology

The decreased popliteal angle and improved passive knee angle at medium-term follow-up in five of the six subjects were as expected. Also the marked shift towards more extended knee angles measured by knee moment-angle characteristics in four of the six subjects was expected. Medium-term improvement in knee angle could not be shown for subject 1 in whom a major adverse event occurred (see S1 Table). Our data and those of previous studies suggest that a more extended knee joint measured by both clinical examination and instrumented hand-held dynamometry can be expected after medial hamstring lengthening [4, 9, 11], at least when no major adverse events complicate recovery and rehabilitation.

Assuming that the ST largely contributes to the knee extension limitations before surgery [16], the improved knee angles towards extension after surgery can theoretically be the result of an increase in ST muscle belly length, which given the parallel fibered architecture of ST implies longer fascicles, and/or an increase in ST tendon length. However, instead of an increase in ST muscle belly length we showed a reduction in muscle belly length. This reduction is most likely due to fascicle shortening by a reduction in the number of sarcomeres in series. A decrease in number of sarcomeres in series after surgery would reduce extensibility of fascicles, which was confirmed by the decrease in muscle belly length change from 0 and 4 Nm. In contrast, ST tendon length was substantially increased, which exceeded the shortening of ST muscle belly length and therefore ST MTU length was increased. It seems that the increased ST tendon length after surgery likely contributed to the increase in knee angle towards knee extension.

At short-term follow-up, the mean slope of the knee moment-angle curve was decreased, suggesting a decrease in stiffness of the structures spanning the knee joint. However, this was only temporary. At medium-term follow-up, there was no difference in slope. As there were no changes in ST muscle belly and tendon lengths back to pre-surgical values, the recurred steeper slope of knee moment-angle curve must be the result of other changes such as (1) length changes of (other surgically) treated muscles (i.e. gracilis and semimembranosus), (2) changes in mechanical properties (i.e. increased stiffness) of hamstring MTUs and/or (3) increased stiffness of extramuscular connective tissue structures (e.g. due to scar tissue formation). Scar tissue of hamstring tendons has also been observed at repeated hamstring lengthening during surgery [9]. The formation of scar tissue may counteract the initial decrease in stiffness and may cause the recurrent stiffness at medium-term follow-up.

### Effect of surgery on joint position during gait

After ST lengthening surgery, all children showed improved knee angles in mid-stance and terminal swing during gait, while effects on hip flexion were variable. The magnitude of increase in knee extension in mid-stance is similar to that reported in previous studies [4, 30]. In addition, the effects of surgery on knee angle in terminal swing are similar to those of previous studies [4, 30]. The biarticular ST is maximally stretched in terminal swing with the hip in maximal flexion and the knee extended [31]. Therefore, lengthening of the biarticular ST may lead to a reduction in passive resistance of ST against hip flexion and knee extension. In both terminal swing and mid-stance, subjects with larger improvements of knee angles (i.e. more extended knee joint) showed larger decreases in hip flexion (subject 3) or unchanged hip flexion angles (subject 2 and subject 4), while subjects with less improvement in knee angles towards knee extension showed an increase in hip flexion (subjects 5 and 6, Fig 6). These relations indicate that the effect of medial hamstring lengthening can differ for knee and hip, with a greater change around the knee (i.e. more change towards knee extension) in most children.

The observation that all subjects in the current study showed an increased anterior pelvic tilt after medial hamstring lengthening, was higher (i.e. 100%) than the percentage that has been reported previously (i.e. 43%) [4]. During the stance phase of gait, an increased anterior tilt occurs when knee joint angles improve towards extension while hip flexion remains unchanged or is increased. Therefore, a decrease in hip flexion (i.e. a more extended hip) would be necessary to compensate for the effects of increased anterior pelvic tilt due to hamstring lengthening. However, after medial hamstring lengthening, ST may not sufficiently contribute to stabilize the hip during stance, because its capacity to generate active force is likely reduced, due to a decrease of PCSA of ST. Also, walking aids may influence pelvic tilt [32]. Three subjects walked with different support during gait analysis after surgery than before (subject 3, subject 4 and subject 6) (S3 Table). These three subjects were the only ones with an anterior tilt greater than normal reference values during mid-stance (Fig 5A). Possibly the change in support affected the pelvic tilt in these subjects in addition to changes in active force generation.

### Clinical implications

A decrease in muscle belly length of ST after surgical lengthening indicates a decrease in length range of active force exertion of ST, while a decrease in ST muscle PCSA suggests a decrease in its force generating capacity. However, previous research has shown that while after hamstring lengthening strength of knee flexor muscles declined initially, it returned back to pre-surgical values nine months after hamstring lengthening [33]. This indicates that other knee extensor and hip flexor muscles (i.e. m. biceps femoris, m. semimembranosus) might compensate for the decrease in PCSA of ST.

Optimal treatment to improve knee angles towards extension in mid-stance should increase knee extension movement and maintain ST muscle belly length as well as PCSA. A longer ST MTU without shortening of muscle belly may be obtained when the ST tendon is lengthened without break-down of of sarcomeres in series. Muscle activation simultaneous with stretch has been suggested to maintain the number of sarcomeres [34]. Resistance training [35] in joint positions in which muscles are strained and/or active-movement training in a stretched position [36] may therefore be required to counterbalance the decrease in muscle belly length and PCSA. In addition, the method of lengthening the ST (Z-lengthening of the tendon vs. tenotomy or aponeurotomy) [37] and the magnitude of lengthening may contribute to the extent by which the muscle belly is strained during stretching exercises as well as during daily activities. Compared to aponeurotomy [37], Z-lengthening of ST tendon, as performed in the current study, may result in less extension of the muscle belly due to a longer and more compliant distal ST tendon. Experimental aponeurotomy in rat m. gastrocnemius medialis has shown to increase optimum muscle length without a decrease in optimal force [38]. Future research should investigate the effects of different lengthening procedures of ST (i.e. z-lengthening of the tendon, (percutaneous) tenotomy and aponeurotomy) on its morphology and relate these to knee joint mechanics, gait and functional outcome in order to improve treatment outcome of SEMLS including medial hamstring lengthening. In addition, different rehabilitation protocols comprising resistance training, stretching and immobilization should be investigated for their effects to counteract atrophy and a shortening of the ST muscle belly. When considering hamstring lengthening surgery to improve gait, it is important to realize the effects that this may have not just on knee kinematics, but also on kinematics of the pelvis and hip. After medial hamstring lengthening, additional treatments to improve hip movement towards extension may be necessary to counterbalance the increased anterior pelvic tilt after medial hamstring lengthening. An increased resistance to stretch of hip flexor muscles (i.e. m. rectus femoris and m. psoas) as well as weakness of hip extensors may contribute to the anterior pelvic tilt. Surgical strategies to reduce hip flexion, such as proximal m. rectus femoris lengthening and/or m. psoas lengthening, have been suggested [39], however with variable success [40–42]. Note, that none of the children included in the current study were indicated for a hip flexor procedure (S1 Table). Next to hip flexor procedures also strengthening of hip extensors [35] and abdominal muscles by training may help to reduce the increased anterior tilt after surgery. In addition, the impact of walking devices on pelvic tilt should be considered.

### Limitations

The number of subjects in this study was low, but this was the maximum number that could be included from both medical centers during the inclusion period of 3.5 years. Assessments took about three hours and could only be performed on the days that children had to visit the hospital for preliminary or control examinations for surgery to burden children and their parents as less as possible. Therefore, subjects were not measured at all initially scheduled time points. A larger study group would be needed for more comprehensive conclusions about the effects of medial hamstring lengthening on overall treatment outcome. However effects of z-lengthening of the ST tendon on muscle morphology seem to be quite consistent and can be noticed reliably even in a small group of children as assessed in the current study.

We assessed morphology of only one muscle that was surgically treated, while the whole intervention included procedures on multiple muscles and sometimes bones. Due to the duration of measurements it was not feasible to additionally assess morphology of other muscles (e.g. semimembranosus or gastrocnemius muscle). However, muscles with different morphologies will respond differently to surgical lengthening of the tendon (as described by a modeling approach [43]) or to aponeurotomy and these effects should be studied in future as they might influence treatment outcome.

In two of the six children, serious adverse events occurred after surgery. Previous studies have shown that peripheral neurological complications and skin problems (as in the current study), as well as other adverse events (e.g. infection, respiratory problems and pain) occur frequently during or after SEMLS in children with SP [44–47]. These adverse events need to be taken into account when SEMLS are indicated and in the interpretation of outcome of surgery.

In the current study two etiologies of SP (i.e. cerebral palsy and hereditary spastic paresis) have been included. Etiology may impact treatment outcome of medial hamstring lengthening, however the sample size was too low to investigate the effect of etiology. Future studies with a larger sample size are warranted to investigate such effects.

The increase in pelvic anterior tilt that we found could also be characteristic for the natural evolution of gait in children with SP. However, studies that longitudinally quantified deterioration in gait of children with cerebral palsy suggest that changes in pelvic tilt and changes in range of pelvic movement in these children are fairly small [48, 49]. Therefore the surgical interventions are likely the major cause of the observed increase in anterior pelvic tilt.

### Conclusion

Medial hamstring lengthening leads to a longer ST tendon, but a shorter and smaller ST muscle belly. The longer tendon seems to contribute to a more extended knee joint during static measurement as well as reduced knee flexion in mid-stance and terminal swing during gait. The extensibility of ST muscle belly after surgery is decreased, likely by a shorter muscle belly. Maintaining of muscle belly length and PCSA may improve treatment outcome of medial hamstring lengthening.

## Supporting information

S1 Table Individual patient information, surgery, adverse events and rehabilitation program. Supplementary table containing all individual information on gender, age, etiology, length, weight, Gross Motor Function Classification System (GMFCS), surgery, adverse events and rehabilitation and/or additional treatment.
(PDF)

S2 Table Individual data knee joint mechanics and muscle morphology. Supplementary table containing all individual data on knee joint mechanics and muscle morphology for all measured time points from which the summary data are presented in the manuscript.
(PDF)

S3 Table Individual data gait kinematics. Supplementary table containing all individual data on gait kinematics and walking devices. Summary data are presented in the manuscript.
(PDF)

S1 Text Inclusion and exclusion criteria. Supplementary information containing information on the exact inclusion and exclusion criteria of the current study.
(PDF)

## References

[1] Novacheck TF. Orthopaedic treatment of muscle contractures, Section 5: Operative treatment In: Gage JR, Schwartz MH, Koop SE, Novacheck TF, editors. The identification and treatment of gait problems in cerebral palsy. 2nd edition London: Mac Keith Press; 2009 p. 445–72.

[2] Rutz E, Baker R, Tirosh O, Brunner R. Are results after single-event multilevel surgery in cerebral palsy durable? Clin Orthop Relat Res. 2013;471(3):1028–38. doi: 10.1007/s11999-012-2766-9 23283676 10.1007/s11999-012-2766-9 PMC3563809

[3] Kay RM, Rethlefsen SA, Skaggs D, Leet A. Outcome of medial versus combined medial and lateral hamstring lengthening surgery in cerebral palsy. J Pediatr Orthop. 2002;22(2):169–72. . 11856923

[4] Dreher T, Vegvari D, Wolf SI, Geisbusch A, Gantz S, Wenz W, et al Development of knee function after hamstring lengthening as a part of multilevel surgery in children with spastic diplegia: a long-term outcome study. J Bone Joint Surg Am. 2012;94(2):121–30. Epub 2012/01/20. doi: 10.2106/JBJS.J.00890 . 22257998 10.2106/JBJS.J.00890

[5] Rodda J, Graham HK. Classification of gait patterns in spastic hemiplegia and spastic diplegia: a basis for a management algorithm. Eur J Neurol. 2001;8 Suppl 5:98–108. . 11851738 10.1046/j.1468-1331.2001.00042.x

[6] Gage JR. Surgical treatment of knee dysfunction in cerebral palsy. Clin Orthop Relat Res. 1990; (253):45–54. . 2317990

[7] Ounpuu S, Solomito M, Bell K, DeLuca P, Pierz K. Long-term outcomes after multilevel surgery including rectus femoris, hamstring and gastrocnemius procedures in children with cerebral palsy. Gait Posture. 2015 doi: 10.1016/j.gaitpost.2015.07.003 . 26260009 10.1016/j.gaitpost.2015.07.003

[8] Adolfsen SE, Ounpuu S, Bell KJ, DeLuca PA. Kinematic and kinetic outcomes after identical multilevel soft tissue surgery in children with cerebral palsy. J Pediatr Orthop. 2007;27(6):658–67. Epub 2007/08/25. doi: 10.1097/BPO.0b013e3180dca114 . 17717467 10.1097/BPO.0b013e3180dca114

[9] Dhawlikar SH, Root L, Mann RL. Distal lengthening of the hamstrings in patients who have cerebral palsy. Long-term retrospective analysis. J Bone Joint Surg Am. 1992;74(9):1385–91. . 1429794

[10] Zwick EB, Saraph V, Zwick G, Steinwender C, Linhart WE, Steinwender G. Medial hamstring lengthening in the presence of hip flexor tightness in spastic diplegia. Gait Posture. 2002;16(3):288–96. . 12443954 10.1016/s0966-6362(02)00022-x

[11] Chang WN, Tsirikos AI, Miller F, Lennon N, Schuyler J, Kerstetter L, et al Distal hamstring lengthening in ambulatory children with cerebral palsy: primary versus revision procedures. Gait Posture. 2004;19(3):298–304. doi: 10.1016/S0966-6362(03)00070-5 . 15125919 10.1016/S0966-6362(03)00070-5

[12] Oberhofer K, Stott NS, Mithraratne K, Anderson IA. Subject-specific modelling of lower limb muscles in children with cerebral palsy. Clin Biomech (Bristol, Avon). 2010;25(1):88–94. Epub 2009/10/20. doi: 10.1016/j.clinbiomech.2009.09.007 . 19836868 10.1016/j.clinbiomech.2009.09.007

[13] Noble JJ, Fry NR, Lewis AP, Keevil SF, Gough M, Shortland AP. Lower limb muscle volumes in bilateral spastic cerebral palsy. Brain Dev. 2014;36(4):294–300. . 23790825 10.1016/j.braindev.2013.05.008

[14] Lampe R, Grassl S, Mitternacht J, Gerdesmeyer L, Gradinger R. MRT-measurements of muscle volumes of the lower extremities of youths with spastic hemiplegia caused by cerebral palsy. Brain Dev. 2006;28(8):500–6. . 16690238 10.1016/j.braindev.2006.02.009

[15] Handsfield GG, Meyer CH, Abel MF, Blemker SS. Heterogeneity of muscle sizes in the lower limbs of children with cerebral palsy. Muscle Nerve. 2016;53(6):933–45. doi: 10.1002/mus.24972 . 26565390 10.1002/mus.24972

[16] Haberfehlner H, Jaspers RT, Rutz E, Becher JG, Harlaar J, van der Sluijs JA, et al Knee Moment-Angle Characteristics and Semitendinosus Muscle Morphology in Children with Spastic Paresis Selected for Medial Hamstring Lengthening. PLoS One. 2016;11(11):e0166401 doi: 10.1371/journal.pone.0166401 . 27861523 10.1371/journal.pone.0166401 PMC5115739

[17] Smith LR, Lee KS, Ward SR, Chambers HG, Lieber RL. Hamstring Contractures in Children With Spastic Cerebral Palsy Result from a Stiffer ECM and Increased In Vivo Sarcomere Length. J Physiol. 2011 Epub 2011/04/14. doi: 10.1113/jphysiol.2010.203364 . 21486759 10.1113/jphysiol.2010.203364 PMC3115830

[18] Fry NR, Gough M, McNee AE, Shortland AP. Changes in the volume and length of the medial gastrocnemius after surgical recession in children with spastic diplegic cerebral palsy. J Pediatr Orthop. 2007;27(7):769–74. Epub 2007/09/20. doi: 10.1097/BPO.0b013e3181558943 . 17878783 10.1097/BPO.0b013e3181558943

[19] Shortland AP, Fry NR, Eve LC, Gough M. Changes to medial gastrocnemius architecture after surgical intervention in spastic diplegia. Dev Med Child Neurol. 2004;46(10):667–73. . 15473170 10.1017/s0012162204001124

[20] Bleck EE. Orthopedic management in cerebral palsy. Philadelphia: J.B. Lippincott; 1987 344 p.

[21] Palisano RJ, Rosenbaum P, Bartlett DJ, Livingston M. Gross Motor Function Classification System Exanded and Revised (GMFCS—E & R). CanChild Centre for Childhoold Disability Research, McMaster University, 2007.

[22] Reimers J. Contracture of the hamstrings in spastic cerebral palsy. A study of three methods of operative correction. J Bone Joint Surg Br. 1974;56(1):102–9. . 4818835

[23] Cave EF, Roberts SM. A method for measuring and recording joint function from the Fracture Clinic of the Massachusetts General Hospital. J Bone Joint Surg. 1936;18:455–65.

[24] Haberfehlner H, Maas H, Harlaar J, Newsum IE, Becher JG, Buizer AI, et al Assessment of net knee moment-angle characteristics by instrumented hand-held dynamometry in children with spastic cerebral palsy and typically developing children. J Neuroeng Rehabil. 2015;12(1):67 doi: 10.1186/s12984-015-0056-y 26272620 10.1186/s12984-015-0056-y PMC4536590

[25] Haberfehlner H, Maas H, Harlaar J, Becher JG, Buizer AI, Jaspers RT. Freehand three-dimensional ultrasound to assess semitendinosus muscle morphology. J Anat. 2016 doi: 10.1111/joa.12501 . 27271461 10.1111/joa.12501 PMC5013067

[26] Kerkum YL, Harlaar J, Buizer AI, van den Noort JC, Becher JG, Brehm MA. An individual approach for optimizing ankle-foot orthoses to improve mobility in children with spastic cerebral palsy walking with excessive knee flexion. Gait Posture. 2016;46:104–11. doi: 10.1016/j.gaitpost.2016.03.001 . 27131186 10.1016/j.gaitpost.2016.03.001

[27] Cappozzo A, Catani F, Croce UD, Leardini A. Position and orientation in space of bones during movement: anatomical frame definition and determination. Clin Biomech (Bristol, Avon). 1995;10(4):171–8. Epub 1995/06/01. . 11415549 10.1016/0268-0033(95)91394-t

[28] Grunt S, van Kampen PJ, van der Krogt MM, Brehm MA, Doorenbosch CA, Becher JG. Reproducibility and validity of video screen measurements of gait in children with spastic cerebral palsy. Gait Posture. 2010;31(4):489–94. doi: 10.1016/j.gaitpost.2010.02.006 . 20304653 10.1016/j.gaitpost.2010.02.006

[29] Kadaba MP, Ramakrishnan HK, Wootten ME. Measurement of lower extremity kinematics during level walking. J Orthop Res. 1990;8(3):383–92. Epub 1990/05/01. doi: 10.1002/jor.1100080310 . 2324857 10.1002/jor.1100080310

[30] Abel MF, Damiano DL, Pannunzio M, Bush J. Muscle-tendon surgery in diplegic cerebral palsy: functional and mechanical changes. J Pediatr Orthop. 1999;19(3):366–75. . 10344322

[31] Cooney KM, Sanders JO, Concha MC, Buczek FL. Novel biomechanics demonstrate gait dysfunction due to hamstring tightness. Clin Biomech (Bristol, Avon). 2006;21(1):59–66. doi: 10.1016/j.clinbiomech.2005.08.014 . 16214274 10.1016/j.clinbiomech.2005.08.014

[32] Krautwurst BK, Dreher T, Wolf SI. The impact of walking devices on kinematics in patients with spastic bilateral cerebral palsy. Gait Posture. 2016;46:184–7. Epub 2016/05/01. doi: 10.1016/j.gaitpost.2016.03.014 . 27131199 10.1016/j.gaitpost.2016.03.014

[33] Damiano DL, Abel MF, Pannunzio M, Romano JP. Interrelationships of strength and gait before and after hamstrings lengthening. J Pediatr Orthop. 1999;19(3):352–8. . 10344319

[34] Van Dyke JM, Bain JL, Riley DA. Preserving sarcomere number after tenotomy requires stretch and contraction. Muscle Nerve. 2012;45(3):367–75. doi: 10.1002/mus.22286 . 22334171 10.1002/mus.22286

[35] Seniorou M, Thompson N, Harrington M, Theologis T. Recovery of muscle strength following multi-level orthopaedic surgery in diplegic cerebral palsy. Gait Posture. 2007;26(4):475–81. Epub 2007/09/15. doi: 10.1016/j.gaitpost.2007.07.008 . 17855096 10.1016/j.gaitpost.2007.07.008

[36] Zhao H, Wu YN, Hwang M, Ren Y, Gao F, Gaebler-Spira D, et al Changes of calf muscle-tendon biomechanical properties induced by passive-stretching and active-movement training in children with cerebral palsy. J Appl Physiol. 2011;111(2):435–42. Epub 2011/05/21. doi: 10.1152/japplphysiol.01361.2010 21596920 10.1152/japplphysiol.01361.2010 PMC3154697

[37] Dagge B, Firth GB, Palamara JE, Eizenberg N, Donath S, Graham HK. Biomechanics of medial hamstring lengthening. ANZ J Surg. 2012;82(5):355–61. doi: 10.1111/j.1445-2197.2012.06030.x . 23305051 10.1111/j.1445-2197.2012.06030.x

[38] Brunner R, Jaspers RT, Pel JJ, Huijing PA. Acute and long-term effects on muscle force after intramuscular aponeurotic lengthening. Clin Orthop Relat Res. 2000; (378):264–73. . 10987002 10.1097/00003086-200009000-00037

[39] Park MS, Chung CY, Lee SH, Choi IH, Cho TJ, Yoo WJ, et al Effects of distal hamstring lengthening on sagittal motion in patients with diplegia: hamstring length and its clinical use. Gait Posture. 2009;30(4):487–91. doi: 10.1016/j.gaitpost.2009.07.115 . 19665381 10.1016/j.gaitpost.2009.07.115

[40] Delp SL, Arnold AS, Speers RA, Moore CA. Hamstrings and psoas lengths during normal and crouch gait: implications for muscle-tendon surgery. J Orthop Res. 1996;14(1):144–51. doi: 10.1002/jor.1100140123 . 8618157 10.1002/jor.1100140123

[41] Morais Filho MC, de Godoy W, Santos CA. Effects of intramuscular psoas lengthening on pelvic and hip motion in patients with spastic diparetic cerebral palsy. J Pediatr Orthop. 2006;26(2):260–4. doi: 10.1097/01.bpo.0000194700.06398.a2 . 16557146 10.1097/01.bpo.0000194700.06398.a2

[42] Mallet C, Simon AL, Ilharreborde B, Presedo A, Mazda K, Pennecot GF. Intramuscular psoas lengthening during single-event multi-level surgery fails to improve hip dynamics in children with spastic diplegia. Clinical and kinematic outcomes in the short- and medium-terms. Orthop Traumatol Surg Res. 2016;102(4):501–6. doi: 10.1016/j.otsr.2016.01.022 . 27050557 10.1016/j.otsr.2016.01.022

[43] Delp SL, Zajac FE. Force- and moment-generating capacity of lower-extremity muscles before and after tendon lengthening. Clin Orthop Relat Res. 1992; (284):247–59. . 1395302

[44] Inan M, Sarikaya IA, Yildirim E, Guven MF. Neurological complications after supracondylar femoral osteotomy in cerebral palsy. J Pediatr Orthop. 2015;35(3):290–5. doi: 10.1097/BPO.0000000000000264 . 25075896 10.1097/BPO.0000000000000264

[45] Lee SY, Sohn HM, Chung CY, Do SH, Lee KM, Kwon SS, et al Perioperative complications of orthopedic surgery for lower extremity in patients with cerebral palsy. J Korean Med Sci. 2015;30(4):489–94. doi: 10.3346/jkms.2015.30.4.489 25829819 10.3346/jkms.2015.30.4.489 PMC4366972

[46] Stout JL, Gage JR, Schwartz MH, Novacheck TF. Distal femoral extension osteotomy and patellar tendon advancement to treat persistent crouch gait in cerebral palsy. J Bone Joint Surg Am. 2008;90(11):2470–84. Epub 2008/11/04. . 18978417 10.2106/JBJS.G.00327

[47] Karol LA, Chambers C, Popejoy D, Birch JG. Nerve palsy after hamstring lengthening in patients with cerebral palsy. J Pediatr Orthop. 2008;28(7):773–6. doi: 10.1097/BPO.0b013e318186bdbb . 18812906 10.1097/BPO.0b013e318186bdbb

[48] Bell KJ, Ounpuu S, DeLuca PA, Romness MJ. Natural progression of gait in children with cerebral palsy. J Pediatr Orthop. 2002;22(5):677–82. . 12198474

[49] Johnson DC, Damiano DL, Abel MF. The evolution of gait in childhood and adolescent cerebral palsy. J Pediatr Orthop. 1997;17(3):392–6. . 9150031

