# Supplementary material for: Outcome of medial hamstring lengthening in children with spastic paresis: A biomechanical and morphological observational study
Source: PLoS One. 2018 Feb 6;13(2):e0192573. doi: 10.1371/journal.pone.0192573 (PMC5800595; doi:10.1371/journal.pone.0192573)
Supplement: S1 Table — Supplementary table containing all individual information on gender, age, etiology, length, weight, Gross Motor Function Classification System (GMFCS), surgery, adverse events and rehabilitation and/or additional treatment. (PDF) [file pone.0192573.s001.pdf]

S1 Table. Individual patient information, surgery, adverse events and rehabilitation program

Subject:<br>Gender<br>Age<br>Etiology<br>Length<br>Weight<br>GMFCS	Surgery	Adverse events	Rehabilitation / additional treatment
1:<br>female<br>10.6 years<br>cerebral palsy<br>146 cm<br>46 kg<br>GMFCS III	Both legs distal medial hamstring lengthening, adductor and psoas release;<br>left leg: hemi-epiphysiodesis	Neuropathic pain after epidural painmanagement; morphine pump<br>GMFCS III=> GMFCS IV	First 6 weeks: immobilization in knee extension brace left leg (23 hours per day); first half year three to four times a week physiotherapy including strengthening, mobilization, pain rehabilitation and hydrotherapy; second half year after surgery two to three times per week physiotherapy including training on parallel bars, transfers training and biking
2:<br>male<br>12.7 years<br>hereditary spastic paresis<br>136 cm<br>27 kg<br>GMFCS III	Both legs distal medial hamstring lengthening and hemi-epiphysiodesis; right leg: exorotating femur osteotomy, endorotating tibia osteotomy and aponeurotic lengthening of triceps surae (Vulpius); left leg: aponeurotic lengthening of triceps surae (Vulpius) and triple arthrodesis foot;<br>After one week of first SEMLS: triple arthrodesis right foot		First 6 weeks: no-weight bearing; immobilization in knee extension brace both legs (23 hours per day); Till half year after SEMLS four times a week physiotherapy including gait training, mobilization, and strengthening; after 12 weeks therapy included two times training in water;<br>Eight months after SEMLS: Botuline toxin A treatment: psoas, adductors, rectus femoris, flexor dig. brevis and flexor dig. longus;
3:<br>female<br>17.2 years<br>cerebral palsy<br>154 cm<br>47 kg<br>GMFCS III	Both legs distal medial hamstring lengthening and supracondylar extension osteotomy; Right leg: aponeurotic lengthening of triceps surae (Vulpius)		First 8 weeks immobilization in knee extension brace both legs (23 hours per day); inpatient rehabilitation nine weeks including gait training, mobilization, and strengthening; Till half year after SEMLS two times a week physiotherapy, then one time.
4:<br>male<br>15.8 years<br>cerebral palsy<br>151 cm<br>53 kg<br>GMFCS II	Both legs distal medial hamstring lengthening three month prior to SEMLS;<br>SEMLS: supracondylar extension osteotomy; patellar tendon shortening; both feet: fusion of calcaneocuboid joints		First 9 weeks: immobilization in knee extension brace both legs (23 hours per day); one hour mobilization and standing exercises; followed by knee extension brace for 2-3 hours and three sessions of physiotherapy every week, including gait training, mobilization and strengthening.<br>After SEMLS: 12 weeks of inpatient rehabilitation.<br>Two years after SEMLS: Rectus release both legs
5:<br>female<br>11.2 years<br>hereditary spastic paresis<br>151 cm<br>46 kg<br>GMFCS II	Both legs distal medial hamstring lengthening and distal femoral anterior guided growth (hemi-epiphysiodesis)	Pain at growth plate at location of fixation plates till half year after surgery	First 12 weeks: immobilization in removable knee extension brace both legs (8 hours per night); combined with five times a week physiotherapy including gait training, mobilization, and strengthening. Till half year after SEMLS two times a week physiotherapy, then one time. Biking at five weeks after SEMLS.
6:<br>male<br>15.2 years<br>cerebral palsy<br>176 cm<br>61 kg<br>GMCS III	Both legs distal medial hamstring lengthening;<br>right leg hemi-epiphysiodesis, exorotating femur osteotomy, endorotating tibia osteotomy; Left leg: hemi-epiphysiodesis, triple arthrodesis foot;<br>After four weeks of first SEMLS: triple arthrodesis right foot	Stage 4 pressure ulcer surgically treated	First 8 weeks: immobilization in knee extension brace both legs (23 hours per day); 6 weeks below-knee cast for mobilization, 8 hours per night knee extension braces, inpatient rehabilitation 12 weeks including gait training, mobilization, and strengthening; Till half year after SEMLS three times a week physiotherapy, then one time. Wheelchair sports training four times a week.
