# Supplementary material for: Outcome of medial hamstring lengthening in children with spastic paresis: A biomechanical and morphological observational study
Source: PLoS One. 2018 Feb 6;13(2):e0192573. doi: 10.1371/journal.pone.0192573 (PMC5800595; doi:10.1371/journal.pone.0192573)
Supplement: S2 Table — Supplementary table containing all individual data on knee joint mechanics and muscle morphology for all measured time points from which the summary data are presented in the manuscript. (PDF) [file pone.0192573.s002.pdf]

S2 Table. Individual data knee joint mechanics and muscle morphology

		Baseline	T1 (10 wk-20 wk)	T2 (5 mo- 9 mo)	T3 (10 mo – 20 mo)	T4 (> 20 mo)
1	Time before/after surgery	12 weeks before	11 weeks after	5 month after	11 month after	23 month after
	ℓ_{femur}	34.9 cm	36.0 cm	35.1 cm	36.2 cm	41.1 cm
	Popliteal angle	70°		70°	70°	55°
	Maximal knee extension	15°		20°	20°	
	θ_{0Nm}	77°	82°	86°	81°	75°
	θ_{4Nm}	50°	41°	60°	67°	52°
	ℓm^{0Nm} / ℓt_{dist}^{0Nm}	29.0 / 13.0 cm		24.4 / 18.2 cm	22.5 / 21.5 cm	22.4 / 28.4 cm
	ℓm^{65deg} / ℓt_{dist}^{65deg}	28.9 / 13.4 cm	26.2 / 18.3 cm	22.7 / 21.6 cm	22.7 / 25.3 cm	22.4 / 28.4 cm
	ℓm^{4Nm}/ ℓt_{dist}^{4Nm}	28.9 / 14.2 cm		24.9 / 22.2 cm	21.2 / 27.2 cm	
	Vol	10.8 cm^3			10.3 cm^3	
2	Time before/after surgery	18 weeks before	20 weeks after	9 month after	12 month after	
	ℓ_{femur}	31.6 cm	31.4 cm	33.2 cm	32.0 cm	
	Popliteal angle	80°	40°	40°	45°	
	Maximal knee extension	45°	/	/	25°	
	θ_{0Nm}	101°	64°	79°	65°	
	θ_{4Nm}	70°	18°	32°	34°	
	ℓm^{0Nm} / ℓt_{dist}^{0Nm}	21.1 / 12.9 cm	18.3 / 20.3 cm	18.5 / 21.8 cm	15.6 / 24.4 cm	
	ℓm^{65deg} / ℓt_{dist}^{65deg}	22.0 / 14.9 cm	18.3 / 20.3 cm	17.5 / 23.6 cm	15.6 / 24.4 cm	
	ℓm^{4Nm}/ ℓt_{dist}^{4Nm}	22.0 / 14.9 cm	15.6 / 26.0 cm	17.5 / 25.6 cm	14.1 / 28.0 cm	
	Vol	32.8 cm^3	17.6 cm^3	17.7 cm^3	16.1 cm^3	
3	Time before/after surgery	6 weeks before	12 weeks after	7 month after	11 month after	
	ℓ_{femur}	33.8 cm	32.8 cm	33.6 cm	32.8 cm	
	Popliteal angle	80°	40°	40°	30°	
	Maximal knee extension	40°	/	/	0°	
	θ_{0Nm}	88°	74°	81°	51°	
	θ_{4Nm}	60°	28°	51°	21°	
	ℓm^{0Nm} / ℓt_{dist}^{0Nm}	23.8 / 15.0 cm		19.2 / 24.2 cm	19.2 / 23.4 cm	
	ℓm^{65deg} / ℓt_{dist}^{65deg}	24.6 / 15.2 cm		19.2 / 24.2 cm	20.4 / 22.6 cm	
	ℓm^{4Nm}/ ℓt_{dist}^{4Nm}	26.2 / 16.8 cm	19.8 / 22.9 cm	19.4 / 25.3 cm	19.7 / 26.6 cm	
	Vol	19.1 cm^3	14.8 cm^3	21.4 cm^3	24.2 cm^3	
4	Time before/after surgery	1 day before	17 weeks after		20 month after	
	ℓ_{femur}	32.6 cm	32.1 cm		33.0 cm	
	Popliteal angle	60°	40°		30°	
	Maximal knee extension	20°	15°		0°	
	θ_{0Nm}	67°	54°		49°	
	θ_{4Nm}	36°	26°		28°	
	ℓm^{0Nm} / ℓt_{dist}^{0Nm}	26.8 / 12.3 cm	19.5 / 21.1 cm		17.0 / 33.0 cm	
	ℓm^{65deg} / ℓt_{dist}^{65deg}	25.0 / 15.3 cm	19.0 / 29.8 cm		16.9 / 33.5 cm	
	ℓm^{4Nm}/ ℓt_{dist}^{4Nm}	28.6 / 13.3 cm	19.8 / 27.2 cm		17.9 / 32.8 cm	
	Vol	36.4 cm^3	17.5 cm^3		8.3 cm^3	
5	Time before/after surgery	6 weeks before		8 month after		
	ℓ_{femur}	38.2 cm		38.1 cm		
	Popliteal angle	70°		50°		
	Maximal knee extension	25°		0°		
	θ_{0Nm}	79°		69°		
	θ_{4Nm}	53°		52°		
	ℓm^{0Nm} / ℓt_{dist}^{4Nm}	23.8 / 16.9 cm		16.8 / 26.1cm		
	ℓm^{65deg} / ℓt_{dist}^{65deg}	25.5 / 17.3 cm		16.8 / 27.1 cm		
	ℓm^{4Nm}/ ℓt_{dist}^{4Nm}	25.7 / 17.6 cm		16.0 / 29.7 cm		
	Vol	42.9 cm^3		8.2 cm^3		
6	Time before/after surgery	9 weeks before			12 month after	24 month after
	ℓ_{femur}	41.1 cm			41.1 cm	41.1 cm
	Popliteal angle	70°			55°	50°
	Maximal knee extension	30°			15°	10°
	θ_{0Nm}	86°			62°	67°
	θ_{4Nm}	68°			42°	50°
	ℓm^{0Nm} / ℓt_{dist}^{0Nm}	30.1 / 19.8 cm			20.3 / 28.2 cm	21.5 / 32.4 cm
	ℓm^{65deg} / ℓt_{dist}^{65deg}	34.0 / 16.1 cm			22.8 / 29.7 cm	21.5 / 30.1 cm
	ℓm^{4Nm}/ ℓt_{dist}^{4Nm}	34.0 / 18.9 cm			23.0 / 31.2 cm	20.9 / 30.7 cm
	Vol	66.9 cm^3			49.8 cm^3	31.6 cm^3

ℓ_{femur}: femur; θ_{0Nm}, θ_{4Nm} knee angle corresponding to 0 Nm and 4Nm net knee moment; ℓm: length muscle belly: tuber ischiadicum to distal muscle tendinous junction; ℓt_{dist}=length of distal tendon. ℓm, ℓt_{dist} are measured at three knee angles (i.e. θ_{0Nm}, 65 degree knee flexion θ_{4Nm})
