# Supplementary material for: Outcome of medial hamstring lengthening in children with spastic paresis: A biomechanical and morphological observational study
Source: PLoS One. 2018 Feb 6;13(2):e0192573. doi: 10.1371/journal.pone.0192573 (PMC5800595; doi:10.1371/journal.pone.0192573)
Supplement: S3 Table — Supplementary table containing all individual data on gait kinematics and walking devices. Summary data are presented in the manuscript. (PDF) [file pone.0192573.s003.pdf]

S3 Table. Individual data gait kinematics

subject		Baseline	T2/T3 (8 mo – 20 mo)
2	Time before/after surgery	18 weeks before	12 month after
	MSt Pelvic tilt	-1,03°	-23,67°
	TSw Pelvic tilt	-0,72°	-23,33°
	MSt Hip angle	37,55°	44,33°
	TSw Hip angle	54,86°	53,33°
	MSt Knee angle	70,10°	37,00°
	TSw Knee angle	72,06°	34,67°
	Walking device*	posterior walker	posterior walker
3	Time before/after surgery	6 weeks before	11 month after
	MSt Pelvic tilt	-21,17°	-38,42°
	TSw Pelvic tilt	-10,79°	-28,65°
	MSt Hip angle	56,75°	45,97°
	TSw Hip angle	66,46°	56,79°
	MSt Knee angle	80,10°	24,48°
	TSw Knee angle	72,04°	25,12°
	Walking device*	holding hand	anterior walker
4	Time before/after surgery	1 day before	20 month after
	MSt Pelvic tilt	-33,54°	-51,79°
	TSw Pelvic tilt	-30,42°	-38,08°
	MSt Hip angle	46,58°	53,37°
	TSw Hip angle	65,58°	68,69°
	MSt Knee angle	38,52°	6,30°
	TSw Knee angle	41,26°	20,63°
	Walking device*	no	posterior walker
5	Time before/after surgery	6 weeks before	8 month after
	MSt Pelvic tilt	-4,40°	-31,31°
	TSw Pelvic tilt	11,70°	-13,48°
	MSt Hip angle	18,89°	30,97°
	TSw Hip angle	34,03°	53,95°
	MSt Knee angle	50,57°	19,55°
	TSw Knee angle	50,71°	33,49°
	Walking device*	no	no
6	Time before/after surgery	9 weeks before	12 month after
	MSt Pelvic tilt	-17,58°	-45,04°
	TSw Pelvic tilt	-12,45	-41,90°
	MSt Hip angle	26,73°	60,05°
	TSw Hip angle	40,03°	77,58°
	MSt Knee angle	39,31°	21,11°
	TSw Knee angle	48,79°	41,80°
	Walking device*	posterior walker	walking stick with four legs

MSt: mid-stance; TSw: terminal swing; *during gait analysis
