# Supplementary material for: Outcome of medial hamstring lengthening in children with spastic paresis: A biomechanical and morphological observational study
Source: PLoS One. 2018 Feb 6;13(2):e0192573. doi: 10.1371/journal.pone.0192573 (PMC5800595; doi:10.1371/journal.pone.0192573)
Supplement: S1 Text — Supplementary information containing information on the exact inclusion and exclusion criteria of the current study. (PDF) [file pone.0192573.s004.pdf]

S1 Text. Inclusion and exclusion criteria

Inclusion criteria for the current study were: (1) a clinical diagnosis of SP due to cerebral palsy or hereditary spastic paresis [24-26], (2) being selected for ST lengthening within a SEMLS or as a single procedure. Indications for surgery were (a) a fixed knee flexion limitation of ≥15º and/or a popliteal angle of ≥60 º and (b) a gait pattern with flexion of the knee in midstance and endorotation-adduction movement of the hips in terminal swing, (3) Gross Motor Function Classification System (GMFCS) [27] level I, II (walking without walking aids) or III (walking with a walking aid), and (4) an age between 6 and 20 years. Patients were excluded if they had interfering treatment and/or had a co-morbidity that could possibly affect walking ability and the tissue properties of the hamstring muscles. We considered as interfering treatment: (1) use of medication that affected neuromuscular properties, (2) treatment with Botulinum toxin A and/or (3) serial casting within 3 months before measurements, as well as any preceding (4) selective dorsal rhizotomy, (5) hamstring muscle surgery, or (6) intrathecal baclofen treatment.
